# Avoidance Learning Across the Menstrual Cycle: A Conceptual Replication

**DOI:** 10.3389/fendo.2020.00231

**Published:** 2020-04-24

**Authors:** Esther K. Diekhof, Sina Korf, Franziska Ott, Carolin Schädlich, Sarah K. C. Holtfrerich

**Affiliations:** Faculty of Mathematics, Informatics and Natural Sciences, Department of Biology, Institute of Zoology, Neuroendocrinology and Human Biology Unit, Universität Hamburg, Hamburg, Germany

**Keywords:** estrogen (17β-estradiol), menstrual cycle, replication crisis in psychology, reinforcement learning (RL), meta-analysis, progesterone and estradiol, reward processing

## Abstract

Hormonal transitions across the menstrual cycle may modulate human reward processing and reinforcement learning, but previous results were contradictory. Studies assessed relatively small samples (*n* < 30) and exclusively used within-subject designs to compare women in hormonally distinct menstrual cycle phases. This increased the risk of sporadic findings and results may have been disproportionally affected by expectancy effects. Also, replication studies are widely missing, which currently precludes any reliable inferences. The present study was intended as a conceptual replication of a previous study [(1), Neuropsychologia 84; *n* = 15]. There, we had observed a reduction in avoidance learning capacity when women were in the high estradiol state of the late follicular phase as compared to the mid luteal phase with enhanced progesterone influence. These results conformed to the idea that estradiol and progesterone may antagonistically modulate dopaminergic transmission as a dopamine agonist and antagonist, respectively. Heightened progesterone in the luteal phase thereby supported the ability to learn from the negative outcomes of one's actions, while the follicular rise in estradiol interfered with this capacity. Here, we re-examined the above described within-subject difference between the follicular and the luteal phase in a between-subjects design. Seventy-five women were tested once with a probabilistic feedback learning task, while being either in the follicular (36 women) or luteal phase (39 women), and were compared for phase-related differences in behavior. Secondly, we combined the new data with data from three previous studies from our laboratory that used the same task and menstrual cycle phases. This meta-analysis included only data from the first test day, free of any biasing expectancy effects. Both analyses demonstrated the consistency of the decline in avoidance learning in the follicular relative to the luteal phase. We also showed that this decline reliably occurred in all of the included samples. Altogether, these results provide evidence for the consistency of a behavioral difference and its apparent association with a transient change in hormonal state that occurs in the natural menstrual cycle. Our findings may also open new avenues for the development of reliable between-subjects test protocols in menstrual cycle research.

## Introduction

There is an ongoing debate about whether menstrual cycle phase related differences in the concentrations of estradiol and progesterone significantly affect human reinforcement learning and reward seeking behavior as well as the associated neural processes. Although previous studies demonstrated a relationship between these hormones and different aspects of human reward processing (2), results showed a high variability and were not always consistent (3). Moreover, most studies in this domain were largely underpowered [*n* < 30; average sample size = 17 women; (3)] and replication studies are currently lacking. It is therefore unclear whether previous observations in humans were a product of the prevailing publication bias in the cognitive sciences or whether they indeed reflected the relatively strong effects of estradiol and progesterone in the mesocorticolimbic dopamine system that are suggested by animal studies.

In female rodents estradiol acts as natural dopamine agonist, which promotes the sensitivity for reward and interferes with the ability to avoid actions that lead to an undesired outcome (4, 5). In contrast, progesterone may partly inhibit dopaminergic transmission, and there is evidence that it can antagonize estradiol's action in the dopamine system (6–8). Progesterone should thus in turn support the ability to avoid actions that lead to a negative outcome (9). In line with the rodent evidence, the neuroimaging study by Diekhof and Ratnayake (1) found that women showed a reduced ability to learn from negative feedback in the high estradiol state of the late follicular phase compared to the mid luteal phase, in which progesterone reached its cyclic maximum. However, their results were based on the data of 15 young women, who were repeatedly tested. A repeated test protocol is not unproblematic, as the task repetition can lead to expectancy or carry-over effects, which may contaminate the experimental effect, one is actually interested in (10–12). Further, such a small sample may preclude the generalizability of the results to a larger population or could even reflect a false positive, sporadic finding. The present study was therefore intended as a conceptual replication of results of Diekhof and Ratnayake (1). The term “conceptual replication,” as we use it here, refers to the repetition of a test of a hypothesis or a result of earlier research work with a different method. This means that the immaterial information focus (i.e., the experimental task performed by the participants) remained the same between studies. In contrast, the material realization of this information differed in line with the experimental idea (i.e., here we intended to replicate a within-subject effect related to changes in hormonal state with a between-subjects design). In contrast, the term “direct replication” refers to the exact repetition of the experimental procedure of a previous study. This can be accomplished by testing a larger repetition sample with the same experimental setup as used in the first study [in our example this would have been equivalent to a within-subject design simply applied to a bigger sample; see Schmidt (13) for discussion and overview].

Here, we assessed the above described difference in avoidance learning capacity between the late follicular and the mid luteal phase (1) in a larger sample and with a between-subjects design, a procedure through which we avoided contamination by repeated testing. For further confirmation of the observed effect, we combined the newly collected behavioral data with those collected in three previous studies from our laboratory that employed the same probabilistic feedback learning task and tested women in comparable menstrual cycle phases. All data for this meta-analysis came from independent observations collected during the first, naïve test day. The meta-analytic data were examined for between-subjects effects associated with menstrual cycle phase. In that way we wanted to evaluate the consistency of the behavioral findings across studies and also intended to compare the results derived from the between-subjects design with the outcome of the commonly used within-subject approach in menstrual cycle research.

## Materials and Methods

### Participants

In total, 93 healthy young women [age [mean ± sem] = 25.2 ± 0.4 years] were tested for this study. They had no current or previous psychiatric or neurological diagnosis, reported to have no history of drug abuse, and did not have any chronic disorder related to the hormone system (e.g., Diabetes, Hashimoto-Thyroiditis, PCO). All reported to have regular menstrual cycles (cycle length between 21 and 35 days), were fluent in German, and had corrected-to-normal vision. Thirty-six women had never used hormonal contraception. The remaining 57 women had taken hormonal contraceptives in the past and reported the month of the last intake. None of them had used hormonal contraceptives within the month preceding the study [average distance between test and last intake [mean ± sem] = 27.7 ± 4.3 months; median distance = 15 months]. Three women had stopped oral contraction <3 months before the actual test, two of them were in the follicular phase on the test day. All subjects gave written informed consent and were paid for participation. The present study was approved by the local ethics committee (*Ethikkommission der Ärztekammer Hamburg*).

### Power Analysis for Determination of Optimal Sample Size

Here, we opted for testing a group of sufficient size to reach an acceptable level of statistical power (≥80%). The sample size for the given project was determined based on the results of Diekhof and Ratnayake (1). There, women were better at avoiding the least rewarded option “B” in the mid luteal phase [mean avoidance frequency ± SD = 77.0 ± 21.1%] compared to the late follicular phase [mean avoidance frequency ± SD = 62.5 ± 16.1%; correlation between paired values = 0.136; Cohen's d = −0.52]. Assuming a power of at least 80%, this behavioral difference translated to an effect size of d_z_ = 0.59 in G^*^Power [(14); please note that in G^*^Power d_z_ = |μ_x−y_| / σ_x−y_, which is somewhat different from Cohen's d = [μ-c]/σ]. In order to achieve the same effect size and a power of 80% at *p* < 0.05 in a between-subjects design, G^*^Power indicated an optimal sample size of 37 women for each test group in the direct comparison of the two cycle phases. For the assessment of the interaction between “*learning preference*” in the reinforcement learning task and “*cycle phase*” a slightly higher number of participants per group was indicated (*n* = 42).

### Post-test Exclusion Criteria

Menstrual cycles are highly variable and thus crucial events, like ovulation, are to a certain extent unpredictable. Cycle phase was therefore determined by a two-step procedure. The test appointment was made based on the onset of menstruation in the given cycle and the expected cycle length based on retrospective information on average cycle length provided by the participant. After the behavioral test took place and the given cycle ended with the onset of the next menstruation, we adjusted the test day to the actual cycle length (actual test day). The actual test day was then standardized to a 28-day cycle for all participants (see Experimental procedure below for standardization formula).

Further, to make our test procedure most similar to the procedures commonly used in within-subject designs, we also decided to exclude participants whose standardized cycle day indicated that they had been tested too late in the follicular phase, when hormone level could have been highly unstable (on the 3 days during and around ovulation). We also excluded participants who were tested when estradiol and progesterone were at their nadir (near the onset or offset the menstrual cycle). These post-test exclusion criteria applied to 16 of the already tested women. Eight of these women were sampled directly before, during or after ovulation, namely at standardized cycle days 13, 14, and 15 of the standard 28-day cycle. Another two women had a positive ovulation test at the time of testing. While the remaining 6 women were either tested at cycle onset (before standardized cycle day 2; *n* = 4) or near its offset (after standardized day 27; *n* = 2). Please note, that based on the pre-definition of time bin, not all of the subjects, who were actually tested on standardized cycle days 13–15 underwent an ovulation test, as this applied only for the predefined time bins 4–6 (see Supplementary Table 1). Another two women did not report the onset of the next cycle and did not reply to our further email inquiries, which also led to their exclusion. Thus, altogether 18 of the 93 women had to be excluded after the behavioral test was completed.

### Experimental Procedure

We planned to test 100 women over the course of 6 months (November 2017–May 2018). Tests were performed under supervision of two female experimenters. Altogether, we tested 93 women during this period, of whom 75 were included in the final analysis after application of the post-test exclusion criteria (see above). Each subject was tested once within one of ten pre-determined time bins that comprised two to four cycle days (Supplementary Table 1). The pre-definition of time-bins was used to schedule the tests of a sufficient number of subjects in an equal distribution across the two cycle phases of interest and to balance testing between the two female experimenters in charge of data collection. The pre-defined test day of a given woman, i.e., the prospective test day, was based on the onset of her menstruation in the present menstrual cycle and the expected cycle length, which was determined from the average length of two previous menstrual cycles. Following the behavioral test and the onset of the next menstruation this information was then adjusted to the actual cycle length of the given test cycle and standardized to a cycle length of 28 days [standardized cycle day = actual test day/ actual cycle length ^*^ length of standard 28-day cycle]. Based on this calculation, we determined that 36 of the 75 women were in fact tested during the follicular phase (standardized cycle days 2–12), while 39 women performed the test in the luteal phase (standardized cycle days 16–26), approximating the optimal sample size as determined by G^*^Power. The result of this calculation was thereby comparable to other counting methods previously used to determine cycle phase, such as the “reverse counting method” [e.g., Puts (15)]. The reverse counting method uses the participant's date of the onset of menstruation of the next cycle and counts back from that date by 14–15 days to retrospectively approximate the date of ovulation. Relative to this ovulation date the current cycle position of the test day is then determined. We also applied this method to our data and found that 35 of the 36 women were classified by the reverse counting method as being in the follicular phase, while the reverse counting method assigned one of our follicular phase women (actual test day = 11; cycle length of given cycle = 25; standardized cycle day = 12) to the ovulation day (ovulation day according to reverse counting method = 11). Since this woman did not have a positive ovulation test before or at the test day and following our counting method was not tested between standardized days 13–15, we kept her original assignment to the follicular phase. Further, 38 luteal phase tests overlapped between our and the reverse counting method. One woman, who had a relatively long test cycle of 40 days, despite reporting regular cycles earlier, was placed in the luteal phase by our counting method (actual test day = 24; cycle length of given cycle = 40; standardized cycle day = 17), while the reverse counting method indicated that she might have performed the test during the late follicular phase (ovulation day according to reverse counting method = 25). Since she was tested in bin 7 (see Supplementary Table 1), she did not perform an ovulation test prior to the test. However, if we excluded her case from the subsequent analyses below, for example in the ANOVA the interaction between “learning outcome” and “cycle phase” remained significant nevertheless (*p* = 0.048) and the effect size remained identical (partial eta^2^ = 0.05). Since otherwise there was no indication that the reverse counting method was somehow superior to our standardization procedure, we kept the cycle phase determined by our method for all women.

Finally, in order to match the two groups of women for various characteristics, they completed a battery of neuropsychological questionnaires and behavioral tests to assess relevant personality characteristics, cognitive capacity as well as mood and premenstrual symptoms. Working memory capacity was measured with the Digit span test and the combined score of forward and backward span. Impulsiveness was measured with the Barratt Impulsiveness Scale (BIS) (16), and color vision discrimination—as an index of dopamine functioning—was measured with the Lanthony Desaturate Panel D−15 [see Colzato et al. (17)]. The Lanthony score was thereby determined according to Geller (18). Current mood was examined with the Multidimensional Mood Questionnaire [MDBF; (19)] and premenstrual symptoms were determined with the Premenstrual Symptoms Questionnaire by Ditzen et al. (20). Altogether, the women in the two cycle phase groups did not differ in age, education level, working memory capacity (Digit span), impulsiveness (BIS score), dopaminergic capacity (Lanthony score), mood (MDBF score), and premenstrual symptoms (PMS score) during the test day.

### Task Description

**Figure 1:**
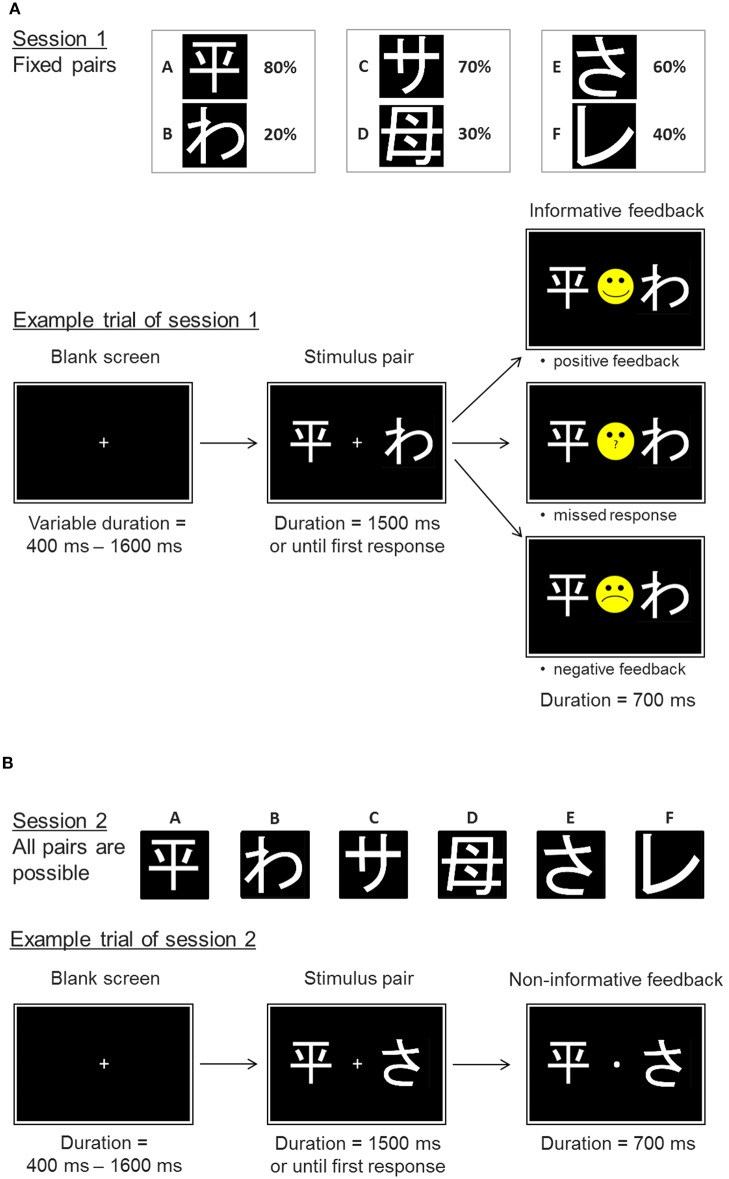
Experimental paradigm. Example trials of the probabilistic feedback task. **(A)** Session 1 represents the learning phase with the fixed stimulus pairs AB, CD, and EF. The probabilities of positive feedback are displayed next to the respective symbols. Example trial: a trial starts with a blank screen delay of variable length. Then the stimulus pair is shown until a response is made or until 1,500 ms have passed without responding. Following the response or the 1,500 ms the participant receives an informative feedback, which either indicates a positive or negative performance outcome (smiley vs. grumpy face) or informs the participant that no response has been made. **(B)** In the transfer phase (session 2) the participants are not only confronted with the original pairs, but also face novel pairs. Responses are no longer followed by informative feedback, but participants just receive a dot indicating that a response has been made.

We used the probabilistic feedback learning task already employed by Diekhof and Ratnayake (1). The task entailed a learning phase, in which participants learned to associate certain stimuli with probabilistic feedback that varied between the stimuli (Figure 1A). During the learning phase (session 1) participants were required to choose the better option from three fixed stimulus pairs (so called pairs “AB,” “CD,” and “EF”) to maximize the incidence of positive feedback (smiley face). The stimuli denoted here as A, B, C, D, E, and F were different hiragana and kanji symbols. Once participants selected one of the symbols from a given stimulus pair, they received direct probabilistic feedback to enforce the different stimulus-feedback contingencies. Pair AB had the highest discriminatory power. Selection of symbol A was “rewarded” with a positive feedback (smiley face) in 80% of selections, while a grumpy face was shown as negative feedback in 20% of selections. In contrast, selection of symbol B yielded a grumpy face in 80% of selections, while only 20% of selections were followed by the positive smiley feedback. The pairs CD and EF yielded a positive feedback in 70:30 and 60:40, respectively. This made symbol A the most often “rewarded” option (best option), while B was the least “rewarded” option (worst option) in the task. Within the other pairs, C and E were the relatively better choices to be made. Before starting the learning phase, participants were instructed to collect as many smiley faces as possible and to avoid the negative grumpy feedback. Unbeknownst to the participants, the combination of the two symbols in the three stimulus pairs was fixed during the 360 trials of session 1. The actual screen location of the two stimuli (left or right) from each pair was pseudorandomly varied as was the sequence of the three stimulus pairs. At the end of the learning phase participants were expected to choose the relatively better options of the three pairs more often than their worse counterparts.

The learning session was followed by a transfer phase (session 2) that also included novel stimulus pairings (e.g., AC, BD, CF). But this time participants did not receive informative performance feedback after their decision [Figure 1B; see also Diekhof and Ratnayake (1)]. Participants were informed about the absence of feedback before session 2 was started, and were instructed to continue with their choices like in session 1, nevertheless. They were not informed about the changes in stimulus pairs. Novel pairs that contained either the symbol A or B allowed us to examine whether subjects rather learned through a preference for the best option A or through avoidance of the worst option B. Preferentially choosing A, the option associated with the highest probability of positive feedback, above all other stimuli in novel pairs is considered as an indicator of the ability to learn from the positive outcome of one's actions. In contrast, an increased avoidance of option B, that was associated with the highest probability for negative feedback, in novel pairs is believed to reflect avoidance learning capacity (21). In all, old and novel pairs were shown 12 times each in a pseudorandomized sequence of pairs and individual screen locations within pairs. The timing of the task remained the same as in the neuroimaging study by Diekhof and Ratnayake (1) (see also Figure 1). The task included the emulation of an fMRI-trigger signal with the repetition time of 2,000 ms as well as a temporal jitter at trial onset.

The transfer phase allowed us to disentangle the ability to learn from the positive outcome of one's actions, here the positive feedback, from the capacity to successfully avoid a negative action outcome, here the negative feedback. It has been assumed that these two aspects of learning may rely on two anatomical routes in the basal ganglia that either promote or inhibit action selection depending on current dopaminergic state (9). These routes may also be subject to the modulation by estradiol vs. progesterone, which may act as a dopamine agonist vs. antagonist, respectively (3). Based on Diekhof and Ratnayake (1), we predicted to find a reduction in the ability to learn from the negative feedback in the high estradiol state of the follicular phase compared to the high progesterone state of the luteal phase. The ability to effectively learn from negative feedback was measured from the percentage of the correct avoidance of the worst option B (“Avoid B” performance) in the novel stimulus pairs of the transfer phase. This required the participant to choose the relatively better stimuli C, D, E, or F from the respective pairs with B, which had all led to a higher incidence of positive feedback than stimulus B in session 1. Conversely, the ability to identify A as the best option was measured by the percentage of selections of A from the novel pairs with A (i.e., AC, AD, AE, and AF) in the transfer phase (“Choose A” performance).

### Collection and Analysis of Saliva Samples

Saliva sample collection followed the common procedure used by our laboratory (1). This included collection of five saliva samples in the morning of the test day. Participants started directly after waking up and provided saliva samples in regular intervals over the course of 2 h. For this they used five 2 ml polypropylene Eppendorf tubes. The samples were frozen at −20°C upon arrival at the laboratory. For the analysis, equal amounts from each of the five samples were combined in an aliquot that was refrozen and then evaluated with a 17beta-Estradiol Saliva ELISA und a Progesterone Saliva ELISA Kit from IBL International (Tecan Group) following the manual provided by the manufacturer. Optical densities were transferred to concentrations with the internet tool https://elisaanalysis.com. The lowest detection level of estradiol in saliva was 2.1 pg/ml, and 3.13 pg/ml for progesterone.

### Description of the Comparison Samples for the Meta-Analysis

For the second analysis, we combined our new behavioral data with previous results from the first test day of Diekhof and Ratnayake (1). In addition, the data from two other unpublished within-subject studies from our laboratory were also included. In all studies women were tested in the late follicular or the mid luteal phase at the first, naïve test day. All studies used the same version of the probabilistic feedback learning task described above, which was always performed first, before any other cognitive tasks included in the different studies. Other study specificities (like daytime or season) were not further considered here. All studies had in common that they assessed healthy young women with a comparable degree of education (on average undergraduate or graduate university education) and age (mean age ± sem = 24.27 ± 0.35 years; age range = 20–30 years), and the pre-test exclusion criteria were identical. All data included here were from the first, naïve test day.

The fMRI study by Diekhof and Ratnayake (1) contributed 15 cases of whom nine were tested in the late follicular phase and six in the mid luteal phase on test day one. The data for this fMRI study were collected by three female experimenters from August 2012 to March 2013. Session 1 of the probabilistic feedback learning task was performed in the MR-scanner, while the subsequent transfer task (session 2) was completed in a secluded test room directly after the participant left the MR-scanner.

The first unpublished study included in the meta-analysis was an EEG study. Originally this study tested women in three hormonally distinct cycle phases (menstruation, late follicular, and mid luteal phase), using a counterbalanced within-subject design. Three female experimenters collected the data between November 2012 and April 2013. Here, we used the data of the first naïve test day. Eight women started the EEG study in the late follicular phase and six were in the mid luteal phase at test day one. Women who started the test protocol during menstruation were not considered for the meta-analysis. Both sessions of the probabilistic feedback learning task were performed in a secluded test room, while the EEG was recorded.

The second unpublished study was designed to assess the influence of menstrual cycle phase on the human transcriptome in peripheral blood and assessed its relation to reinforcement learning. Women completed three repeated tests during menstruation, late follicular, and mid luteal phase in a counterbalanced within-subject design. Data for this transcriptome study were collected by two female experimenters from March to June 2017. Ten women were in the late follicular phase on the first test appointment, while eight started the study in the mid luteal phase. The data of the menstruation test were not included here. In the transcriptome study women arrived with an empty stomach at the test facility between 8 a.m. and 9 a.m., where a blood sample for transcriptome analysis was drawn. After that women had a small breakfast and commenced to the secluded test room, where they completed the probabilistic feedback learning task.

The data from the first test day of the three previous studies were combined with the present data to perform a meta-analysis of all data. Since the three previous studies specifically focused on the late follicular and the mid luteal phase, we decided to include only the data points from the late follicular (*n* = 19) and the mid luteal (*n* = 22) phase of the present study. We also standardized the cycle days from previous studies to a 28-day cycle (see procedure above). This resulted in the following distribution of standardized cycle days in the late follicular phase (mean ± sd): fMRI study = 12.3 ± 2.4; EEG study = 11.4 ± 2.4; Transcriptome study = 13.3 ± 2.3; Present study = 10.2 ± 1.5; All studies combined (*n* = 45) = 11.4 ± 2.3. The standardized cycle days of the mid luteal phase were: fMRI study = 22.5 ± 2.5; EEG study = 21.3 ± 4.8; Transcriptome study = 22.7 ± 2.0; Present study = 22.3 ± 1.8; All studies combined (*n* = 40) = 22.2 ± 2.5.

### Statistical Analysis

The statistical analyses were performed with IBM SPSS Statistics (Version 22). We were primarily interested in the results of the transfer phase (session 2) that reflects overall learning outcome and the difference in punishment sensitivity (Avoid B performance), which had been observed when comparing the two cycle phases in the within-subject design of Diekhof and Ratnayake (1). For this we used a repeated measures two-way ANOVA with the within-subject factor “*learning outcome*” (Choose A and Avoid B performance) and the between-subjects factor “*cycle phase*” (follicular and luteal phase). *T*-tests were used for direct *post-hoc* comparisons. Statistical significance was assumed at *p* < 0.05, two-tailed.

## Results

**Figure 2:**
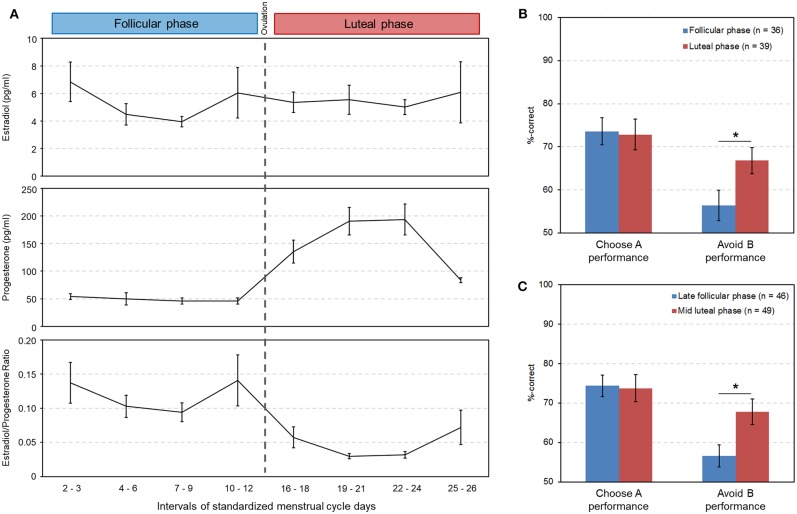
Hormonal changes across the menstrual cycle and their association with reinforcement learning. **(A)** Changes in salivary estradiol, progesterone and in the estradiol/progesterone ratio across the menstrual cycle (data are from independent subjects; cycle days were standardized to a 28-day cycle). Days 13–15, during which ovulation most likely occurred, are not shown here. **(B)** Significantly lower punishment sensitivity in the follicular phase as opposed to the luteal phase in the between-subjects study [*t*_(73)_ = −2.27, *p* = 0.026, Cohen's d = 0.52, 95% CI [lower, upper] = −19.60, −1.27]. **(C)** Significantly lower punishment sensitivity in the late follicular phase compared to the mid luteal phase in the meta-analysis [*t*_(86)_ = −2.62, *p* = 0.01, Cohen's d = 0.56, 95% CI [lower, upper] = −19.60, −2.70]. Significant differences between cycle phases (*p* < 0.05) are indicated with an asterisk.

Group comparisons showed that the two test groups were well-matched for the various characteristics of working memory, personality and mood (Table 1). Salivary hormone concentrations were measured in 28 women of the follicular and in 35 of the luteal phase. Similar to Diekhof and Ratnayake (1) the mean estradiol level did not differ between the follicular and the luteal phase (*p* = 0.97), but there was a significant difference in progesterone (*p* < 0.001) and in the estradiol to progesterone ratio (*p* < 0.001) (Table 1), suggesting different relative contributions of estradiol and progesterone in the two cycle phases (see also Figure 2A for a descriptive overview of the hormonal transitions over time bins).

**Table 1:** Demographic characteristics of the participants.

	**Follicular phase** **Mean ± sem**	**Luteal phase** **Mean ± sem**	***t*-value**	***p*-value (2-tailed)**	**95% CI (lower, upper)**
Age (years)	25.1 ± 0.5 [*n* = 36]	25.2 ± 0.6 [*n* = 38]	−0.16	0.873	−1.69, 1.44
Estradiol (pg/ml)	5.32 ± 0.62 [*n* = 28]	5.34 ± 0.42 [*n* = 35]	−0.04	0.971	−1.47, 1.42
Progesterone (pg/ml)*	**48.74 ± 3.32 [*****n*** **= 28]**	**161.75 ± 14.28 [*****n*** **= 35]**	**−7.71**	**<0.001**	**−142.70**, **−83.32**
Cycle length (days)	29.7 ± 0.5 [*n* = 36]	30.3 ± 0.6 [*n* = 39]	−0.69	0.494	−2.08, 1.01
Duration of menstrual cycle (days)	29.7 ± 0.5 [*n* = 36]	30.3 ± 0.6 [*n* = 39]	0.09	0.925	−0.47, 0.52
Duration of menstrual bleeding (days)	4.9 ± 0.2 [*n* = 36]	4.9 ± 0.2 [*n* = 39]	0.16	0.870	−0.63, 0.75
Standardized cycle day in which women were tested (comparison of all women)*	**7.3 ± 0.6 [*****n*** **= 36]**	**20.9 ± 0.5 [*****n*** **= 39]**	**−17.93**	**<0.001**	**−15.1**, **−12.1**
Standardized cycle day in the comparison of selective parts of the two cycle phases (part of the sample)*	**10.2 ± 0.3 [*****n*** **= 19]**	**22.3 ± 0.4 [*****n*** **= 22]**	**−23.52**	**<0.001**	**−13.1**, **−11.1**
BIS score	63.4 ± 1.7 [*n* = 36]	62.9 ± 1.6 [*n* = 38]	0.21	0.833	−4.2, 5.1
Lanthony score	60.8 ± 1.5 [*n* = 36]	68.5 ± 5.3 [*n* = 38]	−1.41	0.166	−18.74, 3.32
Mood score	90.2 ± 2.6 [*n* = 36]	87.7 ± 2.3 [*n* = 39]	0.745	0.459	−4.28, 9.40
Self-reported stress	2.4 ± 0.2 [*n* = 36]	2.5 ± 0.1 [*n* = 38]	−0.35	0.729	−0.57,0.40
PMS score	20.3 ± 2.5 [*n* = 36]	22.5 ± 2.1 [*n* = 39]	−0.67	0.507	−8.61, 4.29
Digit span (combined forward and backward span)	17.7 ± 0.5 [*n* = 36]	17.4 ± 0.5 [*n* = 39]	0.41	0.684	−1.1, 1.7

Groups may slightly vary in size, as some data could not be acquired from all subjects. *Significant effects (p < 0.05) are plotted in bold and are marked with an asterisk.

Learning outcome of session 1 was comparable between the cycle phases. Accordingly, all participants learned to select the better option from the three pairs AB, CD, and EF with a higher frequency regardless of the cycle phase (all *p* > 0.331; see Table 2). This was similar to Diekhof and Ratnayake (1).

**Table 2:** Learning performance did not differ between the follicular and luteal phase.

**Stimulus pair**	**Follicular phase: selection of better option (%) (*n* = 36)**	**Luteal phase:** **selection of better option (%) (*n* = 39)**	***t*-value (*p*-value)**
Session 1: Pair AB	68.13 ± 3.12	71.36 ± 3.62	−0.67 (0.504)
Session 1: Pair CD	54.87 ± 2.83	59.15 ± 3.30	−0.98 (0.332)
Session 1: Pair EF	55.25 ± 2.38	51.61 ± 2.98	0.95 (0.348)
Session 2: Old Pair AB	75.87 ± 3.90	80.53 ± 4.12	−0.82 (0.416)

*Mean percentage ± sem for the selection of the better option from the respective pairs. Data are from the present between-subjects design*.

The data from the subsequent transfer phase were subjected to a repeated measures, two-way ANOVA with the within-subject factor “*learning outcome*” (Choose A and Avoid B performance) and the between-subjects factor “*cycle phase*” (follicular and luteal phase). Apart from a significant main effect of “*learning outcome*” [*F*_(1, 73)_ = 17.3, *p* < 0.001, partial eta^2^ = 0.19], and the absence of a main effect of “*cycle phase*” [*F*_(1, 73)_ = 1.6, *p* = 0.204, partial eta^2^ = 0.02], we observed a significant interaction between the between-subjects factor “*cycle phase*” and the within-subject factor “*learning preference*” [*F*_(1, 73)_ = 4.0, *p* = 0.049, partial eta^2^ = 0.05]. This was the result of a significant decline of the ability to avoid the option that most often yielded negative feedback (Avoid B performance) in the follicular phase relative to the luteal phase [mean ± sem: follicular phase = 56.38 ± 3.48%; luteal phase = 66.81 ± 3.04%; *t*_(73)_ = −2.27, *p* = 0.026, Cohen's d = 0.52, 95% CI [lower, upper] = −19.60, −1.27]. In contrast Choose A performance remained unaffected by cycle phase [mean ± sem: follicular phase = 73.62 ± 3.12%; luteal phase = 72.86 ± 3.60%; *t*_(73)_ = 0.16, *p* = 0.875, 95% CI [lower, upper] = −8.81, 10.32] (Figure 2B).

A second two-way ANOVA with the same factors, which was however restricted to data points from the late follicular phase, near the pre-ovulatory estradiol peak (standardized cycle days 7–12, *n* = 19), and of the mid luteal phase when progesterone approached its maximum (cycle days 19–24, *n* = 22), confirmed the significant two-way interaction between “*cycle phase*” and “*learning preference*” [*F*_(1, 39)_ = 4.50, *p* = 0.04, partial eta^2^ = 0.10]. The *post-hoc* test showed that avoidance learning was again significantly different between cycle phases [mean ± sem: follicular phase = 54.07 ± 4.65%; luteal phase = 68.48 ± 4.31%; *t*_(39)_ = −2.28, *p* = 0.028, Cohen's d = 0.71, 95% CI [lower, upper] = −27.23, −1.61]. The augmented effect size d however suggests that despite a reduction in sample size the assessment of the late follicular and the mid luteal phase, which should be most distinct in terms of their relative estradiol and progesterone influence, may even enhance discriminatory power in the between-subjects approach. Again, Choose A performance did not differ between cycle phases [mean ± sem: follicular phase = 73.35 ± 4.46%; luteal phase = 70.93 ± 5.04%; *t*_(39)_ = 0.35, *p* = 0.726, 95% CI [lower, upper] = −11.40, 16.23].

**Figure 3:**
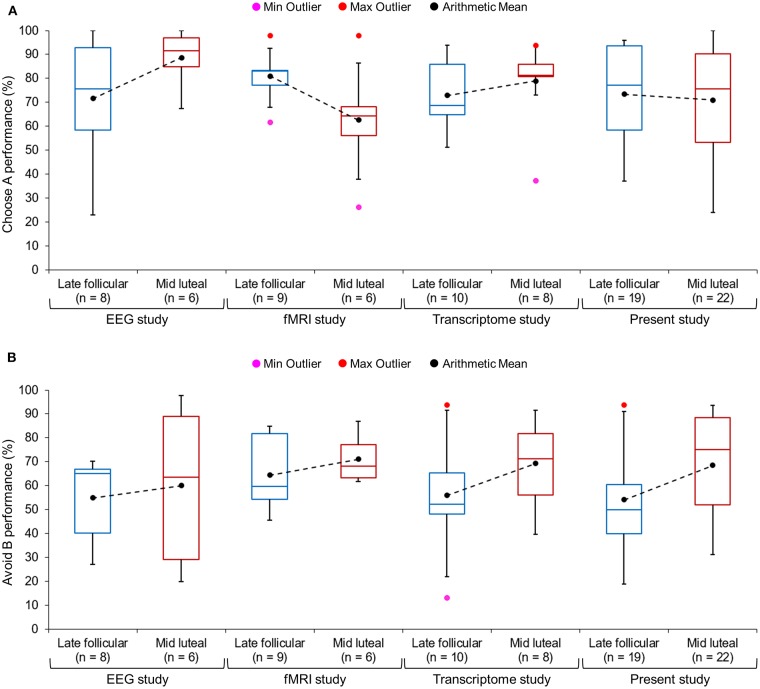
Boxplots of the percentage of **(A)** correct choice of the best stimulus A, and of **(B)** correct avoidance of the worst stimulus B in novel stimulus pairs presented during the transfer phase. The ends of the whisker are set at 1.5*interquartile-range above the third quartile and 1.5*interquartile-range below the first quartile. Only the minimum (Min Outlier) and maximum (Max Outlier) outliers are shown here. We also added the arithmetic mean of the percentage of correct selection for each study and cycle phase to the box plot (see black dots; mean values from the same study are connected by a dashed line). The figure was created with the box plot template by Wittwer (22).

In a second step we performed the two-way ANOVA on the combined data of the transfer task from the three previous and the present study (*n* = 88; n_late follicular phase_ = 49). The associated data from session 1 can be found in Table 3, which shows the comparable learning outcome in both cycle phases. In the transfer phase, a significant interaction between “*cycle phase*” and “*learning outcome*” could be observed [*F*_(1, 86)_ = 4.86, *p* = 0.030, partial eta^2^ = 0.05]. The main effect of “*learning outcome*” was also significant [*F*_(1, 86)_ = 19.79, *p* < 0.001, partial eta^2^ = 0.19], while the main effect of “*cycle phase*” was not [*F*_(1, 86)_ = 2.46, *p* = 0.120, partial eta^2^ = 0.03]. Women were better at avoiding the worst option B during the mid luteal compared to the late follicular phase [mean ± sem: follicular phase = 56.64 ± 2.79%; luteal phase = 67.79 ± 3.23%; *t*_(86)_ = −2.62, *p* = 0.010, Cohen's d = 0.56, 95% CI [lower, upper] = −19.60, −2.70], but demonstrated no difference in choosing stimulus A from novel pairs [mean ± sem: follicular phase = 74.41 ± 2.73%; luteal phase = 73.78 ± 3.39%; *t*_(86)_ = 0.147, *p* = 0.884, 95% CI [lower, upper] = −7.96, 9.23] (Figure 2C). As the sample size of the individual studies that contributed to the meta-data was very small, we only visually inspected the performance data of test day one from each of the four studies (Figures 3A,B). This showed that the difference in Avoid B performance when subtracting the percentage of the late follicular phase from that of the luteal phase (Δluteal-follicular phase) was always negative (mean_Δluteal−follicular phase_: EEG study = −5.15%; fMRI study = −6.71%; Transcriptome study = −13.25%; Present study = −14.42%). In contrast, the delta of Choose A performance varied considerably between studies (mean_Δluteal−follicular phase_: EEG study = −17.04%; fMRI study = 18.29%; Transcriptome study = −5.99%; Present study = 2.42%). This implied that only the ability to avoid a negative outcome appeared to be consistently reduced in the late follicular compared to the mid luteal phase across the four studies from our laboratory, even when comparing independent observations in the two critical menstrual cycle phases.

**Table 3:** Learning performance did not differ between the late follicular and mid luteal phase in the meta-analysis based on the combined data from four independent studies.

**Stimulus pair**	**Late follicular phase: selection of better option (%) (*n* = 46)**	**Mid luteal phase: selection of better option (%) (*n* = 42)**	***t*-value (*p*-value)**
Session 1: Pair AB	69.81 ± 2.69	72.98 ± 2.97	−0.80 (0.429)
Session 1: Pair CD	56.77 ± 2.65	61.10 ± 3.24	−1.04 (0.301)
Session 1: Pair EF	54.26 ± 2.13	55.82 ± 2.59	−0.47 (0.642)
Session 2: Old Pair AB	79.00 ± 3.64	83.31 ± 3.14	−0.89 (0.375)

*Mean percentage ± sem for the selection of the better option from the respective pairs*.

## Discussion

The present study collective was recruited as an independent replication sample, based on which we wanted to evaluate previous results from a study that used the common within-subject design (1). Our study thus constitutes the first attempt of a conceptual replication in the field of menstrual cycle research. Additionally, the present study was intended to demonstrate the feasibility of a between-subjects design in menstrual cycle research. For this purpose, 75 women were tested once with a probabilistic feedback learning task in one of the designated cycle phases. The data from the follicular and the luteal phase were then compared. In a second step, we combined the newly acquired behavioral data with data from three previous studies of our laboratory. Notably, we included only data points from the first, naïve test day of these studies and compared behavior in the late follicular and mid luteal phase. Both analyses demonstrated the consistency of the phase-related difference in avoidance learning and the observed medium effect sizes were in the range of the previously documented within-subject effect. We also showed that, although the actual extent of the reduction in the ability to avoid negative feedback in the high estradiol phase relative to the high progesterone state varied between studies, it occurred in all four studies. Altogether, these results provide evidence for the consistency of a behavioral effect across studies and further underscore the assumption that it may indeed reflect a hormone-related variation in female reinforcement learning ability.

Replications are still rare in the cognitive sciences. Yet, they are necessary to evaluate the implications of fundamental findings for societal and health-related considerations, and to create a solid basis for innovative research (23). Behavioral changes across the menstrual cycle have been assumed to reflect the degree to which estradiol and progesterone influence neural transmission in various cognitive networks, but previous findings were mixed (2, 3). Most results thereby came from within-subject designs that tested only small samples (*n* < 30) and replication studies are currently missing. Therefore, it is possible that many findings were in fact sporadic or might have been disproportionally inflated by involuntary data dredging (24). The present study was an attempt to account for previous shortcomings. We were able to replicate the cycle phase related differences in avoidance learning in a between-subjects design. In that way, our study represents the first conceptual replication of a behavioral within-subject variation (1), which suggests that the observed effect can probably be attributed to changes in hormonal state across the menstrual cycle.

Here, we observed the hypothesized alteration in avoidance learning in women, who were tested only once in a task-naïve state. The behavioral difference was thereby already visible when we compared data that covered the complete follicular and luteal phase (Figure 2B). Diekhof and Ratnayake (1) restricted the two tests to the hormonally more distinct phases of the late follicular and the mid luteal phase. If we did so in our sample, we found that the effect remained significant and the *post-hoc* comparison yielded a slightly higher effect size than the complete sample. This suggests that the finding of reduced avoidance learning in the state, in which the estradiol effect is unopposed by progesterone, i.e., the late follicular phase, compared to the state of increased progesterone influence, i.e., the mid luteal phased, is in fact reliable and can be replicated when comparing two groups of independent subjects in the respective phases of the menstrual cycle.

In addition to that, we combined the present data from the late follicular and the mid luteal phase with the data from the first test appointment of Diekhof and Ratnayake (1) and of two unpublished studies from our laboratory. This meta-analysis comprised 88 data points that were collected by nine different female experimenters between August 2012 and May 2018 and with different ramifications (e.g., in the early morning following a blood-draw for transcriptome analysis, while undergoing EEG-measurement, or in the fMRI scanner). Yet, all studies used the same probabilistic feedback learning task as the first experimental paradigm in the test procedure and assessed young healthy women of comparable age and education. Again, we found that the effect under research was reliable. First, the combined data confirmed the difference in the ability to avoid the least rewarded option between the luteal and follicular phases (Figure 2C), and second, in each of the included studies this difference was negative and thus conformed to the original direction of the within-subject finding (Figure 3B). This latter observation provides further evidence for the inferential reproducibility of the original finding. Amrhein et al. (24) argue that the faulty interpretation of a replication as being non-significant and therefore as representing a contradiction to the original results, only because the *p*-value exceeds 0.05, lets many researchers overlook the fact that *p*-values may rather reflect graded evidence against the null hypothesis. In that way, *p*-values cannot be considered as the main indicator for the reliability of research. This is because *p*-values and significance are hardly replicable, even if the alternative hypothesis is true. Amrhein et al. (24) provide several examples that show that even at a good statistical power of 80%, two studies can be “conflicting,” in that one of the results will be significant and the other will not fulfill the statistical criterion, in one third of the cases, if there is a true effect. A replication can therefore not be interpreted as having failed only because it is non-significant (24). It is often neglected that the replication findings in fact point in the same direction as the original ones, even if the statistical criterion of *p* < 0.05 is not fulfilled. Since the individual samples of our previous studies were too small to statistically compare the data separately, the combined results (Figure 2C) as well as the descriptive finding of a negative Δluteal-follicular phase in all studies (Figure 3A) further support the reliability of the reduction of avoidance learning in the high estradiol state of the follicular phase relative to the luteal phase that was dominated by the effect of progesterone.

What are the implications of the present findings for future research? First, they demonstrate a reliable behavioral effect that is reproducible across different studies, and in a between-subjects comparison, given a matched sample of sufficient size. In that way the present data are consistent with rodent evidence that demonstrated the partly antagonistic effects of estradiol and progesterone on dopaminergic transmission [e.g., (5–8)]. Estradiol may thereby act as a dopamine agonist that promotes reward seeking behaviors, but inhibits the ability to adequately adapt to a punishing outcome. Conversely, progesterone may suppress dopaminergic responses and down-regulates tonic dopamine thus acting in the opposite direction of estradiol (3). Second, the present results may open new avenues for research protocols that examine menstrual cycle effects. By showing that a between-subjects approach may produce comparable results as the common within-subject design, could help to overcome two problems that always accompany within-subject designs: To begin with, the repeated testing of a typical menstrual cycle study can lead to expectancy effects that may contaminate the already small behavioral effects related to changes in hormonal state. Wallen and Rupp (10) showed that the menstrual cycle phase during first exposure to sexual stimuli predicted subsequent interest in sexual stimuli. If women started their test protocol in the high estradiol state of the late follicular phase they showed not only increased interest in the sexually explicit photos there, but this effect was also transferred to the other cycle phases. In contrast, no increased sexual interest in the late follicular phase could be demonstrated, if the women started in any of the remaining cycle phases (10). Leeners et al. (11) noted that “[…] *it is important to recognize that the specific timing of the first test application still introduces a major bias even in counterbalanced test-sequencing designs*.” This is because in one group the practice effect will most likely parallel the actual effect under research (e.g., the influence of estradiol on sexual interest), which could then disproportionally contribute to the expected outcome, or bias processing in the other cycle phases. Therefore, counterbalancing cannot completely wipe out practice effects [see also Leeners et al. (11) for discussion]. Only two studies that assessed changes in stress responsivity across the menstrual cycle intentionally used a between-subjects design and compared the follicular and luteal phase (25) or the early follicular phase and the period around ovulation (26). Maki et al. (25) found that the cortisol stress response was significantly increased in women, who were tested during the follicular phase, and this was also related to the extent of emotional memory impairment women experienced during this phase. Albert et al. (26) reported a reduced distress experience during ovulation that was reflected in altered neural responses. Yet, while in stress-related research between-subjects designs are quite common and also mandatory, since expectations associated with the stress intervention could facilitate modulatory mechanisms like stress coping, to our knowledge the present study is the first between-subjects design employed in the context of reinforcement learning and reward processing.

Moreover, repeated tests at two or even three predetermined cycle phases constitute a logistic challenge. Menstrual cycles tend to show irregularities so that critical phases can be missed, and the daily obligations of a given subject often interfere with test schedules synchronized to individual cycles. Given the limited time frame of research grants this can also considerably limit the sample size. In fact, the average sample size of previous studies in the domain of reward processing is about 17 women [see Diekhof (3) for overview]. Apart from that, lengthy data collection periods may also incur the risk of contamination by other unwanted factors, like seasonal variations in the neuroendocrine response [e.g., Eisenberg et al. (27)]. With regard to the meta-data, we found the largest behavioral difference in the present study which also included the biggest sample. The second largest and numerically almost comparable difference between cycle phases was found in the Transcriptome study, for which the data were collected under the most controlled conditions (Figure 3B). In fact, to keep the blood transcriptome free from contaminating factors (e.g., food intake, circadian, or seasonal influences), women were always tested in the early morning and the study was completed within 3.5 months. In contrast, the two neurophysiological studies tested women whenever time slots were available on the desired test day. Also, data collection was not confined to a certain season. Still, even these latter studies identified the behavioral effect, although it was numerically smaller (Figure 3B). This suggests that a controlled test environment that also considers circadian and seasonal influences on the neuroendocrine response may further support data quality, which again underscores the fact that menstrual cycle research could benefit from test protocols that favor the collection of large samples over a short period of time. Nevertheless, it is also important to point out that between-subjects designs do not only offer advantages, but can produce a number of potential confounds caused by inter-subject variability. The present study tried to closely match participants with regard to education level, age, working memory capacity, impulsiveness, dopaminergic capacity, mood-state, and premenstrual symptoms. All subjects were healthy and did not report any previous or present psychiatric or neurological problems. Yet, it is still possible that the subjects from the two cycle phases differed in an aspect that was not matched here. For example, genetic variability in dopaminergic baseline capacity could represent a significant source of inter-subject variance (28), which was not controlled here. Since the results from the meta-analysis conformed to the present observation, and further replicated an already published within-subject finding, we think that it is very unlikely that an unknown inter-subject aspect determined the group-difference in avoidance learning.

## Conclusion

Taken together, both the present study and the meta-analysis show that our previous within-subject finding can be replicated with a between-subjects design. This does not only support the reliability of the behavioral effect, but also opens new possibilities for future test protocols in menstrual cycle research. The present design circumvents the possible complications caused by repeated testing, since subjects are naïve to the test paradigm. Further, it also does not incur the logistic restrictions of within-subject designs and avoids lengthy periods of data collection. Given sufficient statistical power, i.e., a sufficient sample size that should be pre-determined by a power analysis, close matching of participants, and with careful control of the ramifications that accompany data collection (e.g., by restricting tests to a certain day time or season), future studies should achieve even more valuable contributions to menstrual cycle research by using comparable between-subjects approaches. Within this context, pre-registration of test protocols may further contribute to the reliability of future findings made by menstrual cycle research.

## Data Availability Statement

The datasets generated for this study are available on request to the corresponding author.

## Ethics Statement

The studies involving human participants were reviewed and approved by the Ethikkommission der Ärztekammer Hamburg. The patients/participants provided their written informed consent to participate in this study.

## Author Contributions

ED and SH have contributed in all steps of the research, including development of the experimental design, data collection, preprocessing, and analysis. ED has written the first draft of the paper. FO and CS have contributed in data collection, preprocessing, and analysis. SK has contributed in data analysis. SH, FO, CS, and SK have reviewed the first draft of the paper and provided valuable suggestions for its improvement.

## Conflict of Interest

The authors declare that the research was conducted in the absence of any commercial or financial relationships that could be construed as a potential conflict of interest.
