# Supplementary material for: Avoidance Learning Across the Menstrual Cycle: A Conceptual Replication
Source: Front Endocrinol (Lausanne). 2020 Apr 24;11:231. doi: 10.3389/fendo.2020.00231 (PMC7193994; doi:10.3389/fendo.2020.00231)
Supplement: Supplementary file 1 [file Table_1.DOCX]

**Supplementary Table 1: Classification of pre-defined time bins in the 93 women by means of expected cycle length in relation to the standard cycle of 28 days. Number in bin column indicates the cycle day at which testing in the respective bin started.**

| **Expected Cycle Length**  **(Days)** | **Expected Ovulation (Cycle Day)** | **Bin 1** | **Bin 2** | **Bin 3** | **Bin**  **4** | **Bin**  **5** | **Bin**  **6** | **Bin 7** | **Bin 8** | **Bin 9** | **Bin 10** |
| --- | --- | --- | --- | --- | --- | --- | --- | --- | --- | --- | --- |
| 24 | 11 | 2 | 4 | 6 | 9 | 11 | 14 | 16 | 18 | 20 | 22 |
| 25 | 12 | 2 | 4 | 7 | 9 | 12 | 14 | 16 | 18 | 20 | 23 |
| 26 | 13 | 2 | 5 | 7 | 10 | 13 | 16 | 18 | 20 | 22 | 24 |
| 27 | 14 | 2 | 5 | 8 | 11 | 14 | 17 | 19 | 21 | 23 | 25 |
| **28*** | **14** | **2** | **5** | **8** | **11** | **14** | **17** | **19** | **21** | **23** | **26** |
| 29 | 15 | 2 | 5 | 9 | 12 | 15 | 18 | 20 | 22 | 24 | 27 |
| 30 | 16 | 2 | 6 | 9 | 12 | 16 | 19 | 21 | 23 | 25 | 28 |
| 31 | 17 | 2 | 6 | 10 | 13 | 17 | 20 | 22 | 24 | 26 | 29 |
| 32 | 17 | 2 | 6 | 10 | 13 | 17 | 20 | 23 | 25 | 28 | 30 |
| 33 | 18 | 3 | 6 | 10 | 14 | 18 | 21 | 24 | 26 | 28 | 31 |
| 34 | 18 | 3 | 6 | 10 | 14 | 18 | 21 | 24 | 26 | 29 | 32 |
| 35 | 19 | 3 | 7 | 11 | 15 | 19 | 22 | 25 | 27 | 30 | 33 |
| **Initiation of daily ovulation test** | | n.a. | n.a. | n.a. | 1 to 2 days earlier | 2 to 3 days earlier | 3 days earlier | n.a. | n.a. | n.a. | n.a. |
| **Distribution of all scheduled tests in the time bins based on expected cycle length (n)** | | 11 | 8 | 10 | 8 | 10 | 10 | 11 | 7 | 5 | 13 |
| **Distribution of the final sample (n = 75) across time bins after standardization to 28 days cycle** | | 10 | 8 | 13 | 5 | 6 | 7 | 6 | 5 | 13 | 2 |

*** Standard cycle of 28 days**
